# Exploring commissioners’ understandings of early primary care network development: qualitative interview study

**DOI:** 10.3399/BJGP.2020.0917

**Published:** 2021-06-29

**Authors:** Lynsey Warwick-Giles, Jonathan Hammond, Simon Bailey, Kath Checkland

**Affiliations:** GP, Centre for Primary Care and Health Services Research, Division of Population Health, Health Service Research & Primary Care, School of Health Sciences, Faculty of Biology, Medicine, and Health, University of Manchester, Manchester.; GP, Centre for Primary Care and Health Services Research, Division of Population Health, Health Service Research & Primary Care, School of Health Sciences, Faculty of Biology, Medicine, and Health, University of Manchester, Manchester.; Centre for Health Services Studies, University of Kent, Canterbury.; GP, Centre for Primary Care and Health Services Research, Division of Population Health, Health Service Research & Primary Care, School of Health Sciences, Faculty of Biology, Medicine, and Health, University of Manchester, Manchester.

**Keywords:** clinical commissioning groups, general practice, policy implementation, primary care networks, qualitative research, workforce

## Abstract

**Background:**

Primary care networks (PCNs) are financially incentivised groupings of general practices in the English NHS. Their purpose is to deliver a number of policy goals set out in *The NHS Long Term Plan*. Clinical commissioning groups (CCGs) have a role in their establishment, support, and oversight.

**Aim:**

To explore commissioners’ perspectives on the early development of PCNs.

**Design and setting:**

Qualitative study of CCG staff using telephone interviews.

**Method:**

Semi-structured interviews were carried out with 37 CCG employees involved in PCN establishment. Interviewees were asked about local PCNs’ characteristics, factors shaping development and form, activities to date, challenges and benefits, and their CCGs’ relationship with PCNs. Interviewee responses were summarised within a matrix and analysed thematically.

**Results:**

Three meta-themes were identified: the multifaceted role of the commissioner, tensions between PCN policy and locally commissioned services, and engaging the broader system. Interviewees reported that the policy potentially favours those PCNs working from a ‘blank slate’ and does not sufficiently account for the fact some GP practices and wider system organisations have been doing similar work already. The prescriptive, contractual nature of the policy has led to local challenges, trying to ensure that local good practices are not lost during implementation. Interviewees also considered an important part of their work to be protecting PCNs from the weight of expectations placed on them.

**Conclusion:**

CCGs are well placed to understand the complexities of local systems and to facilitate PCNs and working practices between wider system partners. It is important that this local role is not lost as CCGs continue to merge and cover larger geographical populations.

## INTRODUCTION

There is a perceived crisis in primary care in the English NHS: GPs are leaving the profession at an earlier age than previously or are choosing to work part-time, and there is a lack of newly trained doctors entering general practice.^1^^–^2 Alongside this, demands on the NHS are growing as a result of an increasingly elderly, multimorbid population, and funding is failing to keep pace.^3^ *The NHS Long Term Plan*^4^ set out a number of proposals to address these problems, including the introduction of primary care networks (PCNs) to support GP practices and increase integration with wider system partners.

Policy guidance suggested that PCNs would be geographically contiguous groupings of GP practices usually covering a patient population of 30 000–50 000.^5^ PCNs are being established using a contractual mechanism, a voluntary add-on (or directed enhanced service) to the existing General Medical Services contract, which governs GP services. This add-on contract was negotiated between NHS England and the British Medical Association representing GPs, and it provides additional funding for participation; the employment of new staff under an Additional Roles Reimbursement Scheme; offering extended hours; a performance incentive scheme; and funding for a clinical director for each PCN. Roles covered by the Additional Roles Reimbursement Scheme include clinical pharmacists, social prescribers, physician associates, and others such as paramedics and advanced practitioners. These new workers will be employed across PCNs to relieve pressure on practices. The incentives for GP practices to become involved are thus strong and most GP practices opted to join a PCN in July 2019.^6^

Although PCNs had some discretion in terms of their formation, they required the approval of their local clinical commissioning group (CCG), the body with responsibility for commissioning primary care services, and NHS England. GP practices were given some leeway in deciding both their configuration and the content of an agreement between member practices, which included internal governance processes, a lead practice (a nominated practice to hold the PCN bank account), and arrangements for receiving and distributing funding.^7^ The result has been considerable variation in the size and population coverage of PCNs, with many outside the official target of a population size of 30 000–50 000 people.^8^

PCN development has not been entirely smooth. In return for the additional investment, from April 2020, PCNs were expected to deliver a set of service specifications, covering five areas of work: structured medicines reviews and optimisation; enhanced health in care homes; personalised care; supporting early cancer diagnosis; and anticipatory care for vulnerable patients. However, draft specifications, published in December 2019,^9^ were strongly opposed by GPs and their representative organisations,^10^ with concerns cited about the increased workload involved in meeting the specifications.

**Table:** How this fits in

Primary care networks (PCNs) in the English NHS are new, financially incentivised collaborations between general practices organised via an add-on to the contract GPs have with government. Clinical commissioning groups (CCGs) have had a role in authorising PCNs, as well as supporting their development and operation, often through the provision of additional management support. In some local areas, the contractual approach and stipulations of the policy have slowed down and damaged existing collaborative initiatives. PCNs need to work together with other providers; CCGs are well suited to support this work. However, the supporting role lacks any formality and funding. This article outlines the contribution of CCGs in supporting PCN development.

Further negotiations with the British Medical Association led to significant amendments, including the phasing of the introduction of the specifications, a reduction in associated performance requirements, additional funding, increased reimbursement for all roles from 70% to 100%, and additional payments for providing care in care homes.^11^

In March 2020, the COVID-19 pandemic further delayed the introduction of the service specifications and the introduction of the proposed performance incentive scheme.

This article reports the findings of a national interview study with CCG staff, conducted to better understand how PCNs are constituted and how CCGs are supporting their development. It shows how CCGs have played an important role in the creation and early operation of PCNs, and illustrates the complexities faced in implementing the Network Contract Directed Enhanced Service alongside existing working practices and commissioning arrangements.

This study highlights some issues of key relevance to both local policy implementation and the further development of national policy.

## METHOD

This study is part of an ongoing longitudinal mixed-methods project (July 2019 to July 2023) comprising four work packages: exploring national policy objectives;^12^ telephone interviews with CCG staff to explore how they are supporting PCN development (August to December 2019); case studies of PCNs in five different CCG areas; and quantitative analysis of PCN development and outcomes.^8^

This article focuses on the second of these work packages and reports data from 37 semi-structured qualitative interviews with CCG staff across England who have a role in commissioning or supporting the development of PCNs in their geographical area (roles are director of commissioning, head of primary care, accountable officer, director of place, local care director, and director of transformation). Qualitative interviews explored the role of commissioners and the local implementation of national policy. This work package complemented the quantitative work that took place at the same time. CCGs were purposively selected to provide geographical coverage (Table 1); however, recruiting responders from the London region proved difficult. Prospective interviewees were approached by email, informing them of the research team, the funders of the project, and the purpose of the research. A participant information sheet, outlining the study in more detail and what their involvement would entail, was provided. Responders then made contact with the research team via email to organise a suitable interview time.

**Table 1.** Geographical coverage of clinical commissioning groups (CCGs)

**Geographical area**	**Number of CCGs interviewed**
East of England	6
North West	8
North East and Yorkshire	10
South West	3
Midlands	4
London Region	1
South East	5

The topic guide (Box 1) focused on PCN characteristics; factors shaping their development and form; activities to date; challenges and current and potential perceived benefits; and CCG relationships with PCNs. The study’s central concern was to understand the factors affecting how PCNs were forming and this informed the research questions. The topic guide was piloted with three sites to ensure that the questions were understandable and that the guide would capture the information required. The questions were developed from the authors’ reading of the policy documents, the wider literature, and informal discussions with staff at CCGs, NHS England, and the Department of Health and Social Care.

**Box 1.** Topic guide for telephone interviews

To start can you just explain to me your role within the CCG and your involvement with PCNs? **Local PCNs** How have PCNs been established? *(Flat practice network/lead provider/GP federation–provider entity/super practice as a network, non-GP provider (community trust) employer model)* How many PCNs are within your CCG footprint? How many patients in the largest PCN and smallest PCN? Why have the PCNs been established in this way? *(History / GP federation/geography …)* As a CCG did you reject any PCN applications? *(If so, why?)* Are the clinical directors of local PCNs GPs? *(If not, what job role, and why not a GP?)* Have patients had any role in the development or operation of your PCN? Have any members of staff been recruited using the Network Contract DES within any of your PCNs? *(clincal pharmacist & social prescriber)* What factors have enabled and supported the establishment of PCNs? *(max. 3)* What issues have you faced when trying to establish PCNs? (*max. 3 — 100% coverage/data sharing)* **PCN work and wider organisational involvement** How are GP practices working together? What domains of work are they focusing on? *(What influenced the choices made?)* Are networks working with other organisations? *(If so, at what level: neighbourhood, place, or system level? Get examples from each level where available)* **Intended benefits of PCNs** What are the intended benefits of PCNs for patients? What are the intended benefits of PCNs for practices? What are the intended benefits of PCNs for the CCG? How are outcomes being measured and by whom? **Commissioning and contracting mechanisms** What commissioning arrangements do you have in place as a CCG to support PCNs? *(Is the support to help with their development or for longer term?)* Were any financial incentives put in place to help with PCN development, in addition to the DES? *(From NHS England and/or the CCG?)* What kind of organisation holds the DES contract for most PCNs and how was this decided? What contract mechanisms as a CCG are you using to support collaborative working? *(DES/LES, etc.)* Are NHS England involved with the commissioning of PCN? (If so, explain …) Are NHS England involved with the contracting of PCN? (If so, explain …)

*CCG = clinical commissioning group. DES = Directed Enhanced Service. LES = Local Enhanced Service. PCN = primary care network.*

Telephone interviews, lasting 30 minutes on average, were conducted by two experienced qualitative, health policy researchers and were audio-recorded. Verbal consent was taken before the interview and audio-recorded. A framework analysis approach was employed.^13^ One author summarised responses to each question to populate a matrix using spreadsheet software, and codes were developed from these responses. This process took place alongside data collection. When data saturation was reached, with the same issues being highlighted by participants, no additional interviews were scheduled. Codes were then formulated into categories, which were tested against the authors’ understandings of salient aspects of the policy derived from documentary analysis and findings from the other work packages of the project. This process was accomplished using NVivo (version 12). All authors collaboratively developed the meta-themes below.

## RESULTS

The interviews generated rich data concerning the establishment and form of PCNs, alongside the complexities and issues faced when trying to implement the PCN policy locally. For the purpose of this article, three meta-themes are presented that encapsulate most comments from interviewees: the multifaceted role of the commissioner, tensions between PCN policy and locally commissioned services, and engaging the broader system.

All CCG staff reported numerous factors that had influenced how PCNs had formed. The most common influences that were discussed were historical working relationships, local geography, and the role of the local medical committee (the local statutory representative body for GPs) of the British Medical Association. Responders emphasised that, although PCNs are a new policy initiative, GPs working collaboratively in specific geographical footprints has been happening in many areas for some time (for example, practice-based commissioning and GP federations).

### Theme 1: The multifaceted role of the primary care commissioner

CCG staff identified themselves as having a number of roles that they needed to fulfil when implementing the PCN policy locally; these included supporting the development of PCNs, protecting PCNs from expectations, and coordinating the PCN policy into existing programmes of work.

#### Support and protection

Although GP practices are financially incentivised to join PCNs, the financial entitlements do not provide designated monies for PCN management. This has meant that the staff time and resources for management support have been commonly provided by CCGs to ensure that general practice staff have been able to understand the policy, prepare themselves for the registration procedure, and develop themselves into a PCN.

Twenty-six CCGs reported that they had allocated some of their staff time through their CCG primary care teams to work alongside PCNs through a variety of different mechanisms, including PCNs being added to the primary care team’s portfolio of work, providing workshops to disseminate the policy and facilitate local conversations between PCNs, and drawing together local clinical directors. In the short term, four CCGs had also agreed to second some of their staff to PCNs to provide skills that PCNs were perceived to be lacking, such as finance and management. However, CCG staff were concerned about the sustainability of these arrangements for PCNs and CCGs.

Most CCG staff reported that they helped shape the formation of PCNs locally, ensuring they were geographically contiguous, and in line with policy guidelines. Many CCG staff spoke of having ‘honest and open’ conversations with PCN staff to ensure that PCN development issues had been addressed before registration and applications were not rejected by the CCG. CCG staff liaised with other organisations and entities in the broader system to ensure that, for example, community services were aware of PCN developments, and that PCNs were engaging with the relevant integrated care system or sustainability and transformation partnership (STP). An Integrated Care System [ICS] is a partnership between local organisations, with the responsibility of meeting health and care needs within a geographical area. STPs are a precursor to ICSs, and are partnerships of NHS providers, commissioners, and local authorities, planning health and care delivery for their geographical area.

CCGs were naturally well placed within the system to interact with other system partners because of existing working and commissioning relationships developed over a number of years. CCG staff reported that this position in the system was important in supporting the development of PCNs to ensure they were aligned with the rest of the system.

Some CCG staff had concerns that the expectations being placed on PCNs through central policy initiatives were too extensive and could lead to professional backlash. CCG staff reported that, if the work required was too onerous, general practices would refuse to sign up to the Directed Enhanced Service for the following financial year. CCG staff explained how they were trying to protect their PCNs from such expectations:

*‘It has all come too quickly —* [PCNs have been] *asked to put in place this new entity, employ people, develop new services — it is too much.*’

(IDNS1.14 [ID number survey 1, 14th person interviewed])

*‘We have been shoulder to shoulder* [with the local medical committee] *on how to support PCNs and shield them from the heat as being the solution to everything.’*

[IDNS1.31]

One CCG staff member described PCNs under pressure from too many expectations from organisations across the system:

*‘PCNs are like Buckaroo* [a children’s game] *: people keep piling work on them and everyone wants a bit of them. We need a reality check or we will kill them before they start.’*

[IDNS1.19]

Nine CCG staff described PCNs as being seen as the *‘nirvana’* or *‘cavalry’*; PCN working was perceived to be an opportunity to solve all the problems that the NHS had historically faced without any real evidence of how such things would be achieved. Staff from four CCGs suggested that they felt they needed to protect their local PCNs from the pressures and expectations being placed on them, pushing back on occasion to NHS England and local organisations when they deemed the pressures to be too great for PCNs:

*‘We have been shoulder to shoulder on how to support PCNs & shield them from the heat as being the solution to everything. We have spent lots of time managing up to NHSE* [NHS England] *. We want to ensure that they* [PCNs] *are strong before we start asking too much of them.’*

(IDNS1.31)

#### Coordination

When implementing the PCN policy locally, 10 commissioners discussed the complexities associated with integrating the PCN policy into existing streams of work. This meant that CCG staff had to work to ensure that the new, more prescriptive requirements did not disrupt existing relationships, which were felt to have been successful:

*‘Having* [local integration initiatives] *in place made it more complicated. We weren’t starting with a blank sheet, we had to think about how this would all come together*. *’*

[IDNS1.2]

Staff from five CCGs suggested that the PCN policy had caused local distraction; primary care development was seen as something separate from the local system for a time, because general practices were focusing on the Directed Enhanced Service, to ensure that they were meeting the contractual requirements, rather than collaborating with the wider system.

General practice staff needed time to understand what being a PCN meant and how they wanted to form themselves:

*‘We were further ahead* [than this policy] *; we had arrangements in place, working … in integrated groups. The PCN contract caused lots of distraction for a number of months … it has taken us backwards.’*

[IDNS1.34]

The added complexity faced locally, in some areas, was associated with programmes of work and service delivery, which were broader than general practice and primary care. For example, CCGs had locality and neighbourhood programmes of work focusing on an integration agenda with other system organisations.

The implementation of the PCN policy and the geographical contiguity element of the policy was found to be restrictive in some areas, as it did not allow for recognition of other partnerships and in some instances caused local tensions. This has meant that a substantial amount of CCG time has been spent ‘knitting together’ pre-existing local schemes with PCNs.

### Theme 2: Tensions between PCN policy and locally commissioned services

Responders said that the introduction of PCNs had taken local commissioners and practices by surprise, resulting in a need to re-evaluate existing commissioning decisions and work programmes. Staff from four CCGs stated that, if they had been aware of the policy direction earlier, they would have made different local commissioning decisions recently:

*‘The GP contract is agreed at national level — it is hard for localities to understand the direction of travel. We had made decisions on our* [local service standards] *and the next month the DES* [Directed Enhanced Service] *came out and we may have made different decisions if we would have known what was coming*. *’*

[IDNS1.12]

As a national ‘one size fits all’ contractual approach, the Network Contract Directed Enhanced Service and the new service specifications created duplication across a number of programmes of work for some CCGs. For example, some CCGs had already established local collaborative groups of GPs, with funded positions for a clinical lead. Others had already funded social prescribers and clinical pharmacists locally. These CCGs have had to spend some time reconciling the PCN policy with existing local commissioning arrangements, identifying duplications and potential double-funding problems. The policy was felt by those interviewees to be of more benefit to commissioners who had not been proactive in developing new ways of working in primary care:

*‘Because of historical integrated working, we identified a need and funded mental health practitioners in general practice. The DES* [Directed Enhanced Service] *is prescriptive on roles which has meant we* [CCG] *have had to work through how we will afford the workforce that was in place before the DES*. *’*

[IDNS1.34]

Many CCG staff explained that they have been working on primary care development for some time in their role as (co-) commissioners of primary care services. They described identifying local population needs and recruiting different professional roles to address those needs. For example, a large proportion (13) of CCG staff reported that they had been working with social prescribing organisations for some time. The PCN Additional Roles Reimbursement Scheme had significant implications for these CCGs, because it was a requirement of the Directed Enhanced Service that, to be eligible for the reimbursement for new roles, staff employed had to be new and in addition to existing staff. This meant, for instance, that if the CCG had already established a social prescribing service for their practices, they would only be eligible for the additional funding if they appointed additional staff, and this caused confusion and some practical problems in one CCG area:

*‘We have had to re-deploy social prescribers in areas where the CCG can afford to commission them; otherwise, we wouldn’t be able to draw down the additional resource. We had 15 social prescribers who were on recurrent schemes because we knew money was coming from PCNs. We didn’t realise that by doing it in advance we would be penalised. Practices where the social prescribers were working are having to recruit afresh* . *’*

[IDNS1.19]

### Theme 3: Engaging the broader system

The objectives of the PCN policy are multifaceted; PCNs have been established to create a more collaborative general practice, promote inter-organisational place-based care, and to support and shape the system.^12^ Commissioners suggested that the GP-centric contractual approach to PCN development failed to facilitate the engagement of elements of the local system beyond general practice. The policy frames PCNs as an opportunity to engage and influence the collaborative entities at a broader ‘system’ level (integrated care systems or sustainability and transformation partnerships), with clinical directors representing the voice of local primary care. However, realising this aspiration in practice was considered problematic.

Staff from 10 CCGs spoke of the complexities involved, which included deciding whether all clinical directors needed to attend all relevant integrated care system or sustainability and transformation partnership meetings, and establishing collaboration and governance arrangements so that clinical directors could represent each other and speak with one voice:

*‘We are trying to ensure that their voices are heard but we need to think about the value of clinical time. We are working on a network of leads; they don’t all have to go to everything.’*

[IDNS1.24]

In order to achieve this, PCNs needed mechanisms for consulting their membership and deciding agreed positions. CCG staff described that these things were not simple to accomplish. Some reported that their local PCNs were not yet engaged at the wider system level because GPs did not feel they understood what happened at the system level or why it was relevant to them:

*‘There are negotiations at the moment at STP* [sustainability and transformation partnership] *level to ensure that the PCNs all have a seat at the leadership table. The PCNs themselves are not sure whether they should be there — I think that is about their immaturity. It will change over the next 12–18 months.’*

[IDNS1.32]

Staff from 10 CCGs reported that they saw it as an important part of their role to liaise between PCNs and the wider system, as there was no formal structure to ensure that the system worked effectively with local-level organisations such as PCNs:

*‘The CCG is making the STP* [sustainability and transformation partnership] *link to PCNs. There is an STP-wide meeting with clinical directors but the focus is on integration at a local level. It is where the PCNs believe they can make a difference.’*

[IDNS1.11]

At the time the telephone interviews took place, PCNs were in their early establishment phase and were primarily focused on general practice. CCG staff acknowledged that cooperation between PCNs and their local community service providers would be very important in the future, particularly in relation to delivering the proposed service specifications. Two CCGs had established local arrangements whereby community services were ‘wrapped around’ PCNs. In other areas, geographical boundaries for PCNs had caused local tensions with other providers. Staff from two CCGs acknowledged that they were unsure how their local PCNs and community services were going to function in practice together.

CCG interviewees described how in the short term their primary focus was internal, to support the development of relationships between practices, as it was believed that was a prerequisite for longer-term success. However, they also acknowledged that, as commissioners, they would have an important role in the future in brokering relationships between PCNs and other community providers.

## DISCUSSION

### Summary

The policy establishing PCNs using a centrally negotiated contractual mechanism tied to the core GP contract brings with it a number of complexities. Interviewees reported that CCGs (which co-commission primary care services) had been required to reconcile the top-down contractual changes with existing local primary care support and development mechanisms. For those CCGs where such programmes were most advanced, the PCN policy represented a particular challenge, which some believed had slowed their progress and damaged existing initiatives. As commissioners of primary care services, CCG staff have had a role in supporting the establishment of PCNs and mediating between practices to ensure smooth implementation of comprehensive coverage; providing managerial and administrative support to PCNs that have neither the resources nor capability to do this; reconciling the new contractual requirements with existing commissioning plans; protecting PCNs from perceived excessive expectations and demands from other parts of the system; brokering relationships with other providers in their local area; and supporting PCNs to engage at system level with integrated care systems or sustainability and transformation partnerships.

PCNs are provider organisations, and there is therefore an important role for a body with planning responsibility that can maintain an overview of the local landscape of provision. The General Medical Services contract and associated network Directed Enhanced Service contract do not account for the full range of primary medical care services that need to be commissioned, and it is important that the core and add-on contracts align with existing and planned local service plans and developments. PCNs need to be supported to work together and with other providers, and proactive management and planning are required to ensure that the provision of care meets local needs. CCGs, as commissioners of primary care with a good understanding of their local primary care providers, are ideally suited to fill this role. Future iterations of PCN policy need to engage proactively with CCGs to ensure that policy development meets the needs of local areas and complements local service developments.

### Strengths and limitations

The strength of this study is that it offers some of the first empirical evidence of the issues arising as PCNs were established. Qualitative interviews were undertaken with CCG primary care leads across England, and data saturation was reached, suggesting that the important issues arising had been captured. However, the data were collected at a single point in time and thus represent a snapshot of PCN development. Interviews were carried out before the draft service specifications for PCNs were released; had it been possible to repeat the interviews, this study would likely have gathered additional interesting data about how the specifications were received by PCNs and how CCGs had engaged in the consultation process. Data were not collected from PCN leads, whose perspectives on the roles of CCGs in PCN development may have been different. PCN policy is developing rapidly, and the COVID-19 pandemic has put additional pressure on all parts of the NHS. The impact of these pressures will be captured in the next phase of the research.

### Comparison with existing literature

PCNs are new organisations so there is no existing literature on the topic. However, there is a long history of collaborative working between GP practices, including running collective audits,^14^ providing out-of-hours care,^15^ and undertaking commissioning activity.^16^ Such initiatives have been shown to increase GPs’ wellbeing,^17^ and studies have shown that collaborations between GPs as providers need flexible and enabling managerial support from the commissioning authority.^18^^,^^19^ Studies of primary care commissioning have demonstrated the need for detailed local knowledge and a strong understanding of how primary care is delivered on the part of commissioners, as well as strong relationships between commissioners and providers.^20^^,^^21^ Existing literature thus supports this study’s conclusions about the importance of the CCG’s role in supporting PCN development.

### Implications for practice

The formal guidance surrounding PCNs has been focused on GP practices, with a heavy emphasis on negotiating interpractice agreements and on the minutiae of the rules surrounding contractual payments. This is understandable, given the core role of GP practices in forming PCNs, and is an inevitable consequence of the choice of a nationally negotiated contractual mechanism for implementing the policy. However, this has led to something of a vacuum in guidance relating to the role of CCGs in supporting PCNs. This study has shown that CCG staff take this role seriously, and are keen to provide the support that PCNs need to become established and develop their role. Ongoing policy envisages PCNs as a neighbourhood nucleus around which other services coalesce;^22^ supporting this process will require CCGs to act as brokers of relationships, mediators of disputes, and providers of development and management support. Following on from *The NHS Long Term Plan*,^4^ many CCGs have recently merged, creating significantly larger commissioning organisations.^23^ As CCGs scale up and lose staff, there must be a concern that local, historical knowledge about the system and local trusting relationships and the local links that will be needed for successful collaboration across provider organisations will be lost.

The next phase of PCN development will see PCNs working internally and with other providers to deliver an increased range of services. Ensuring that these services dovetail with and complement existing extended services will be another important role for primary care commissioners. The interplay between national contract negotiation and locally determined service need will require careful management.
